# Photo‐Electro‐Thermal Textiles for Scalable, High‐Performance, and Salt‐Resistant Solar‐Driven Desalination

**DOI:** 10.1002/advs.202400623

**Published:** 2024-06-19

**Authors:** Duo Xu, Can Ge, Ze Chen, Zhixun Zhang, Qian Zhang, Tao Chen, Chong Gao, Weilin Xu, Jian Fang

**Affiliations:** ^1^ College of Textile and Clothing Engineering Soochow University Suzhou 215123 China; ^2^ State Key Laboratory of New Textile Materials and Advanced Processing Technologies Wuhan Textile University Wuhan 430200 China; ^3^ National Engineering Laboratory for Modern Silk Soochow University Suzhou 215123 China

**Keywords:** desalination, electric wire, photo‐electro‐thermal, solar‐driven evaporation, textile manufacturing

## Abstract

Solar‐driven interfacial evaporation is an emerging desalination technology that can potentially relieve the freshwater scarcity issue. To obtain high and continuous evaporation rates for all‐weather, chemically engineered structural materials have been widely explored for simultaneous photothermal and electrothermal conversion. However, many previously reported fabrication processes involve poor integration and considerable energy loss. Herein, a scalable photo‐electro‐thermal textile is proposed to enable high efficiency, long‐term salt rejection, and solar‐driven desalination. Specifically, the photo‐electro‐thermal yarns with a core (commercial electric wire)‐shell (polypyrrole‐decorated Tencel) structure realize the integration of electrothermal and photothermal conversion. The wrapping eccentricity of 1.53 mm and pitch of 3 T cm^−1^ for the electric wire are rationally regulated to achieve a high surface temperature of over 52 °C at a 3 V DC input. As a result, exceptional and stable evaporation rates of 5.57 kg m^−2^ h^−1^ (pure water) and 4.89 kg m^−2^ h^−1^ (3.5 wt.% brine) under 1 kW m^−2^·radiation with a 3 V input voltage are realized. Practical application shows that the textiles can achieve high water collection of over 46 kg m^−2^ d^−1^ over the whole day of operation. The constructed photo‐electro‐thermal textile‐based evaporator provides an effective method for commercial and scalable photo‐electro‐thermal conversion to achieve high‐performance and salt‐resistant solar‐driven desalination.

## Graphical abstract

A composite braiding evaporator is fabricated with electric wire and polypyrrole‐decorated Tencel for efficient desalination. The middle electric wire with customizable wrapping pitch and wrapping eccentricity enables optimized electrothermal conversion efficiency, heat distribution uniformity, and thermal transfer consistency. The optimized evaporator enables long‐term operation even in high‐concentration saline over a whole‐day operation.

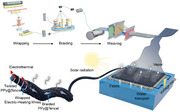

## 1 Introduction

**Figure 1:**
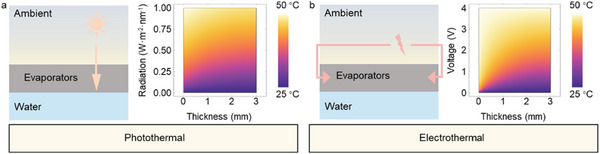
Proposed functions and simulated properties of the ESSG system. The thermal energy of ESSG systems under various a) solar radiation and b) input voltages. The dominant thermal energy caused by a reasonable input voltage matches that of maximal solar radiation.

Solar steam generation (SSG) is a green, cost‐effective, and sustainable solution for water desalination and purification. However, the continuity, stability, and efficiency of SSGs are difficult to maintain due to phenomena such as alternating day and night as well as variable weather and climate.^[^ ^1^, ^2^, ^3^ ^]^ Although several SSGs using auxiliary energy sources of wind power have been reported, these SSGs generally have discontinuous and unstable desalination from undesirable environmental energy harvesting.^[^ ^4^, ^5^, ^6^ ^]^ The electrothermal coupled solar steam generation (ESSG) system with a low voltage input is a low‐cost, reliable, and efficient platform for achieving stabilized and controllable temperature regulation for improving interfacial evaporation yield.^[^ ^7^, ^8^, ^9^ ^]^ The schematics in **Figure** **1** demonstrate that thermal energy is dominated by solar radiation (0 to 1 W m^−2^ nm^−1^) and input voltage (0–4 V), resulting in increased surface temperature and further water vaporization. A similar surface temperature is achievable with a rational input voltage (≈3 V) compared with the thermal energy from the maximal solar radiation (1 W m^−2^ nm^−1^) (Figure S1, Supporting Information). Thus, benefiting from the accessibility, affordability, and stability of electricity, electrothermal conversion might be the perfect candidate to be implemented with highly efficient steam generation.^[^ ^10^ ^]^

Recently, burgeoning developments in ESSG systems have offered a promising strategy to realize continuous and stable interfacial evaporation.^[^ ^11^, ^12^ ^]^ State‐of‐the‐art separated ESSG systems combine with external solar cells or solar panels to store the generated electricity during the daytime to assist in the evaporation of weak radiation conditions.^[^ ^13^, ^14^ ^]^ However, the non‐portability, weather reliance, and energy conversion losses of separate devices have limited their development.^[^ ^15^ ^]^ In the aspect of integrated ESSG systems, these devices directly embed the electrothermal components (wire/sheet/plate) into the device and assist evaporation by a direct current (DC) power supply.^[^ ^16^ ^]^ However, the majority of integrated ESSG research only elaborates on the phenomenon that the coupling of electrothermal can achieve all‐weather operation. The in‐depth investigation and analysis of optimizing the construction to enhance the electrothermal evaporation efficiency and durability need to be further explored.^[^ ^17^, ^18^ ^]^ In addition, the salt accumulation on the evaporator over long‐term desalination not only compromises energy conversion but also blocks the water/steam transport channel.^[^ ^19^, ^20^ ^]^ Salt rejection strategies including Janus evaporator, hydrophilic diffusion, mechanical washing, and salt discharge have been widely developed for durable desalination.^[^ ^21^, ^22^ ^]^ However, most ESSG devices cannot be applied for high‐concentration desalination due to the imbalance in brine circulation and salt nucleation rate caused by electrothermal coupling.

**Figure 2:**
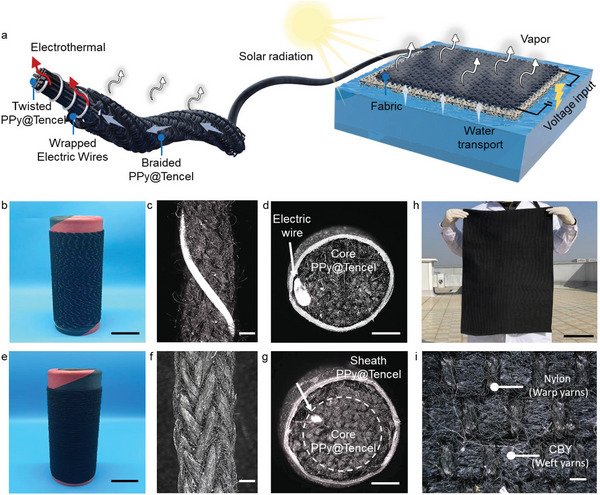
Fabrication and structural characterization of the CBEF evaporator. a) Schematic drawing of the ESSG process and the regulation of energy conversion and brine circulation. b) Digital image (scale bar: 3 cm), c) radial optical image (scale bar: 1 mm), and d) cross‐sectional optical image (scale bar: 1 mm) of the composite bilayer yarn. e) Digital image (scale bar: 3 cm), f) radial optical image (scale bar: 1 mm), and g) cross‐sectional optical image (scale bar: 1 mm) of CBY. h) The digital image of scalable large‐size CBEF (scale bar: 20 cm). i) Optical image of CBEF (scale bar: 2 mm).

In this work, a composite braiding electrothermal fabric (CBEF) enables continuous and stable desalination with boosted electrothermal conversion efficiency is presented through scalable, practical, and universal braiding technology.^[^ ^23^, ^24^ ^]^ These yarns with core‐sheath structures consist of core‐layer polypyrrole (PPy)‐decorated Tencel yarn (PPy@Tencel), middle‐layer commercial electric wire (nickel–chromium alloy), and sheath‐layer PPy@Tencel (**Figure** **2**a). The core‐layered Tencel substrate is employed for a rapid water supply, and the outer‐layered decorated PPy endows a strong photothermal response‐ability.^[^ ^25^, ^26^ ^]^ The structural distribution of middle‐layered commercial electric wire is regulated by wrapping pitch and eccentricity to optimize the electrothermal performance of composite braiding yarns in the radial and axial directions, respectively. The core‐layered PPy@Tencel can provide strong capillary force for sufficient brine advection‐diffusion, resulting from the fiber micropores as well as the interconnected fibrous network. The electrothermal conversion of CBEF is managed to achieve a balance between brine circulation and energy input, thus ensuring optimal evaporation efficiency and desalination durability. Hence, our outstanding ESSG rate of 5.57 kg m^−2^ h^−1^ under 3 V DC input and 1 kW m^−2^ (1 sun) radiation is realized. A stable evaporation rate of 4.89 kg m^−2^ h^−1^ in 3.5 wt.% brine is obtained without salt deposition over 24 h of desalination. The average outdoor desalination energy consumption is only ≈1.29 kW h m^−3^ in conventional saline over the whole day operation. The evaporator remains intact, clean, and stable after consistent operation in 10 wt.% brine without salt crystallization, and its competitive desalination energy consumption is calculated as 1.61 kW h m^−3^ even in high‐concentration saline.

## 2 Results and Discussion

### 2.1 Fabrication and Structural Characterization

The polypyrrole (PPy) decorated Tencel (PPy@Tencel) and commercial electric wire are selected for multifunction integration (Figure S2, Supporting Information). Among them, PPy@Tencel is simultaneously applied as a core layer and sheath layer for water circulation and photothermal conversion, and the middle commercial electric wire is wrapped around the core PPy@Tencel for electrothermal performance optimization. As illustrated in Figures S3 and S4 and Table S1 (Supporting Information), industrial‐scale wrapping, braiding, and weaving technology with convenience, practicality, and universality is applied for the fabrication of composite braiding electrothermal fabric (CBEF) with a certain thickness of ≈5.259 mm.^[^ ^24^, ^27^ ^]^ First, PPy is decorated onto the hydrophilic Tencel to obtain PPy@Tencel through in situ polymerization, while the yarn surface becomes rough after polymerization, and the increased specific surface area facilitates solar absorption (Figure S5, Supporting Information). As shown in Fourier transform infrared spectroscopy (FTIR) spectra (Figure S6, Supporting Information), the peaks of pristine Tencel ≈3340, 2886, and 1030 cm^−1^ are attributed to the ‐OH stretching vibration, C─H asymmetrical stretching vibration, and C─O stretching vibration, respectively.^[^ ^28^ ^]^ Hydrogen bonds and van der Waals forces can be formed between hydrophilic groups and water molecules, which facilitates the water adsorption and migration within Tencel.^[^ ^29^ ^]^ The hydrophilic groups are vital for water supply and transport. After PPy polymerization, the characteristic bands ≈1520 and 1025 cm^−1^ are ascribed to the stretching vibrations of C═C and C─N. Abundant conjugated π bonds can excite electrons and form π─π* transitions at almost every wavelength of the solar spectrum.^[^ ^30^, ^31^ ^]^ Heat will be released when the excited electron relaxes back to its ground state for interfacial evaporation.^[^ ^32^, ^33^ ^]^ The infrared emissivity of CBEF is calculated as 98.24% across 2–15 µm, showing stable energy transfer characteristics (Figure S7, Supporting Information). The generated heat is confined within CBY to achieve lower heat loss due to the dense core‐sheath structure.^[^ ^34^ ^]^

Second, multistranded PPy@Tencel successively converges at the braiding point, is wound onto the bobbin, and is mounted on the designated spindles (Figure S3b, Supporting Information). The reciprocating movement between different disks facilitates the interconnection between the PPy@Tencel yarns. Third, the core PPy@Tencel is guided and fed into a hollow spindle with a tension controller (Figure S3c, Supporting Information). The electric wire is helically wrapped over the core PPy@Tencel at high speed to prepare the large‐scale bilayer composite yarn (Figure 2b). Figure 2c,d shows that the helix spacing and angle changes by the varied relative positions between the electric wire and multistranded PPy@Tencel can be regulated by the feeding speeds and wrapping speeds. Structural engineering is applied for electrothermal enhancement by the optimal wrapping pitch and wrapping eccentricity. Fourth, the sheath PPy@Tencel is secured to the outside disc, and the fabricated composite yarns are wound onto a central bobbin and fed through a pretension device. The sheath PPy@Tencel is wrapped around the double‐layer composite yarns in both clockwise and counterclockwise directions to fabricate composite braiding yarn (CBY) with a diameter of≈2.5 mm (Figure 2e). When braided by sheath PPy@Tencels consisting of multistranded wound yarns, the inner PPy@Tencel and electric wire can be tightly wrapped for a robust structure, as shown in Figure 2f,g. The dry surface temperature of the fabricated CBY with a 10 cm length rapidly reaches over 54 °C after 5 min under a 3 V direct current (DC) voltage (Figure S8, Supporting Information) due to the outstanding electrothermal capacity of the electric wire.^[^ ^35^ ^]^

Furthermore, the scalable large‐size (90 × 60 cm) CBEF is subsequently woven with nylon and CBY conveniently and practically (Figure 2h,i). Nylons are applied as tensional warp threads through the loom heddle with a designed pattern, and CBYs are inserted as weft threads into the tensional warp to form a plain structure (Figure S3d, Supporting Information). The solar absorption of CBEF across 280–2500 nm is measured as 94.7%, which is a significant enhancement compared to the pristine Tencel (Figure S9, Supporting Information). The PPy particles can convert solar energy into internal energy to realize heat localization in the fibrous network through electron excitation and relaxation.^[^ ^36^ ^]^ The surface wetting property is investigated by the water contact angle (WCA) test. The water droplet on the CBEF surface is rapidly infiltrated within 300 ms, which confirms its excellent hydrophilicity (Figure S10, Supporting Information). The fiber micropores endow a strong capillary force, which pumps water and promotes meniscus thin‐film evaporation.^[^ ^37^, ^38^ ^]^ The water‐wicking rate of CBEF is over 3.5 g g^−1^, which ensures a rapid water supply from hydrophilic PPy@Tencel (Figure S11, Supporting Information).

### 2.2 Electrothermal Conversion Optimization

**Figure 3:**
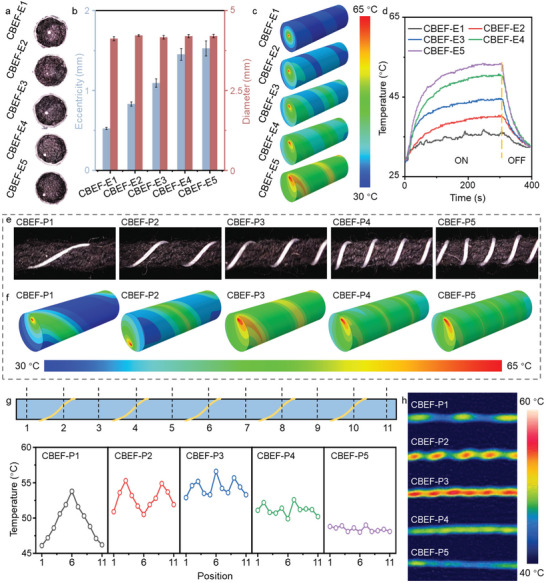
Electrothermal conversion of CBEF evaporator. a) Optical image of CBEF‐Es warping eccentricity regulation. b) The warping eccentricity and diameter of CBEF‐Es. c) Simulation of electrothermal conversion and energy distribution of CBEF‐Es with voltage input. d) Time‐dependent dry temperature variations of CBEF‐Es samples with 3 V DC input. e) Optical image of CBEF‐Ps warping pitch regulation. f) Simulation of electrothermal conversion and energy distribution of CBEF‐Ps with voltage input. g) The stable dry temperature recording at different positions of CBEF‐Ps with 3 V DC input. h) The average stable dry temperature over the whole‐yarn section with 3 V DC input.

The electrothermal conversion performance of CBEF can be optimized by regulating the wrapping eccentricity and wrapping pitch of the electric wire in CBY. Specifically, the wrapping eccentricity is regulated by modifying the position of the electric wire relative to the core PPy@Tencel in the cross‐section (**Figure** **3**a). For CBEF‐Es with a fixed total amount of PPy@Tencel (fixed diameter), the gradually increasing eccentricity implies that the electric wire is shifted from the center to the edge area (Figure 3b). When the number of sheath yarns is increased from 8 to 16 strands, the eccentricities of CBEF‐E1, CBEF‐E2, CBEF‐E3, CBEF‐E4, and CBEF‐E5 are measured as 0.53, 0.83, 1.09, 1.45, and 1.53 mm, respectively. The simulation is carried out to obtain a comprehensive understanding of the electrothermal conversion process (Figure 3c). From the simulated results, it is clear that the surface heat distribution is dramatically affected by electrothermal efficiency. The surface temperature of CBEF‐Es with larger eccentricity rises rapidly because the energy generated by electrothermal conversion can be rapidly transferred to the sheath layer for interfacial heating (Figure S12, Supporting Information). For CBEF‐Es with small eccentricity, the generated heat is mainly concentrated in the restricted interior zone. Based on the simulation analysis, the surface temperature variation is recorded in Figure 3d with thermocouples under a 3 V DC voltage supply. The heat distribution gradients of CBEF‐Es result from the experiments coincide with the finite element method (FEM) results. The temperature drops dramatically when the DC power is cut off, which demonstrates the rapid electrothermal response of the CBEF. As a comparison, the corresponding stable temperature of the composite yarn is only ≈42 °C with a 3 V DC input if the electric wire acts as the core layer (Figure S13, Supporting Information). It can be concluded that the compact multilayer CBY structure ensures the tunability and energy conversion efficiency of the CBEF (Figure S14a, Supporting Information). Therefore, CBEF‐Ps are further optimized on the foundation of CBEF‐E5.

Similarly, the wrapping pitch regulation is realized by modifying the wrap density of the electric wire over the core PPy@Tencel in the radial direction (Figure 3e). The wrapping pitches of CBEF‐P1, CBEF‐P2, CBEF‐P3, CBEF‐P4, and CBEF‐P5 are set as 1, 2, 3, 4, and 5 T cm^−1^, respectively. The wrapping pitches of electric wires can be controlled by the regulation of feeding speeds and wrapping speeds. From the simulated results in Figure 3f, the energy generated through electrothermal conversion is mainly concentrated around the electric wires. Therefore, a rational distribution of the electric wires is essential for electrothermal efficiency enhancement. The electric wires are more densely distributed as the wrapping pitch decreases. As a result, the distribution of the heat generated by the electrothermal conversion is more consistent and intense. However, the extremely small wrapping pitch implies that the resistance of the electric wires is excessively high, which compromises the electrothermal efficiency (Figures S14b and S15, Supporting Information).

According to the simulation analysis, the temperature distribution at different locations of CBEF‐Ps is recorded in Figure 3g with thermocouples under a 3 V DC voltage supply. Overall, the stable surface temperature of CBEF‐P3 with a moderate wrapping pitch is over 54 °C at 3 V DC input, which is significantly higher than that of the other CBEF‐Ps (Figure 3h; Figure S16, Supporting Information). In addition, the heat distribution over the whole yarn segment is very uniform and smooth, which coincides with the simulation results. The optimized CBEF‐P3 possesses a sensitive and immediate electrothermal response ability with a varied DC voltage supply. As shown in Figure S17 (Supporting Information), the dry surface temperature exhibits step‐like and wave‐like fluctuations as the voltage supply rises/falls and switches on/off, respectively. In addition, CBEF‐P3 demonstrates outstanding long‐term durability (over 3000 s) and cyclic stability (over 100 cycles), which is essential for practical efficient ESSG (Figure S18, Supporting Information). Consequently, an optimized CBEF‐P3 with an eccentricity of 1.53 mm and a wrapping pitch of 3 T cm^−1^ is selected for further investigation of its photo‐electro‐thermal steam generation performance.

### 2.3 Electrothermal Coupled Solar Steam Generation System

**Figure 4:**
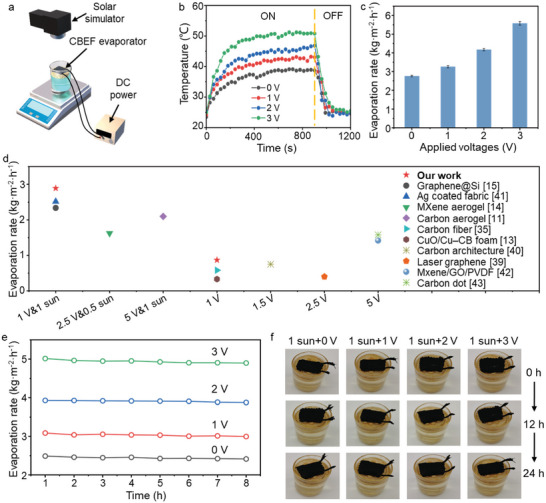
Electrothermal coupled solar steam generation performance of the BCEF evaporator. a) Schematic of the ESSG testing device. b) Time‐dependent wet temperature variations of BCFF‐T samples under 1 kW m^−2^ illumination and 3 V DC input. c) Comparison of the evaporation rate of CBEF‐P3 under 1 kW m^−2^ illumination with varied voltage inputs. d) Comparison with recent studies on ESSG systems. e) The desalination rate comparison of CBEF‐P3 under 3.5 wt.% brine with varied voltage inputs and 1 kW m^−2^ radiation. f) Digital images showing the surface of CBEF‐P3 during the desalination process with varied energy inputs.

The experimental device of the ESSG system is shown in **Figures** **4**a and S19 (Supporting Information). The electric wires are connected to the DC power to generate heat through electrothermal conversion, and the PPy@Tencel is exposed to solar radiation to generate heat through photothermal conversion. CBEF is placed on floated foam and aluminum foil is used to reflect excess radiation. Bulk water is supplied by the ends of CBEF for continuous and rapid evaporation (Figure S20, Supporting Information). The mass variations during ESSG are recorded continuously at 30 s intervals with an electronic balance. The evaporation mass change of CBEF‐Es under 3 V DC input is recorded in Figure S21a (Supporting Information). The evaporation rate improves sequentially with increasing eccentricity, which is consistent with the gradient of the surface temperature distribution. CBEF‐E5 exhibits the best evaporation rate due to concentrated heat utilization. Therefore, CBEF‐Ps are further optimized based on CBEF‐E5. The evaporation rates of CBEF‐P1, CBEF‐P2, CBEF‐P3, CBEF‐P4, and CBEF‐P5 under 3 V DC input are measured as 2.21, 2.55, 2.71, 2.41, and 2.28 kg m^−2^ h^−1^, respectively (Figure S21b, Supporting Information). CBEF‐P3 with a moderate wrapping pitch balances the heat generation and energy distribution, thus yielding an optimized evaporation performance. When 1 kW m^−2^ radiation is further applied, the ESSG performance of CBEFs demonstrates a significant enhancement with the assistance of photothermal conversion. The evaporation rate of CBEF‐P3 under 3 V DC input and 1 kW m^−2^ radiation is up to 5.57 kg m^−2^ h^−1^ (Figure S22, Supporting Information). Overall, the evaporation rate is significantly enhanced after applying CBEF with/without solar radiation (Figure S23, Supporting Information).

The wet surface temperatures of CBEF‐P3 under different DC inputs and solar radiation are continuously recorded with a thermocouple and infrared camera. Under the dark experiment, a stable wet surface temperature of over 35 °C under 3 V DC input is achieved after several minutes (Figure S24, Supporting Information). The surface temperature dramatically increases after weak solar radiation (0.5 kW m^−2^) is applied because of the excellent electrothermal/photothermal coupling effect (Figure S25, Supporting Information). After 5 min of varied DC input and 1 kW·m^−2^ radiation, the stable wet surface temperatures reached 42.6, 45.2, and 50.8 °C under 1, 2, and 3 V DC input, respectively (Figure 4b). When the DC power and solar simulator are cut off, the surface temperature of CBEF‐P3 rapidly drops to the initial state, demonstrating the outstanding electrothermal/photothermal response and electrothermal adaptability at different voltages. Correspondingly, the coupling effect of electrothermal/photothermal methods to accelerate evaporation is significantly enhanced with increased DC input and solar radiation (Figure 4c). Specifically, the evaporation rate of CBEF‐P3 increases from 0.25 (0 V, 0) to 5.57 (3 V, 1 kW m^−2^) kg m^−2^ h^−1^ with enhanced electrothermal/photothermal conversion. The evaporation performances of CBEF with different sizes under varied solar radiation and DC inputs are listed in Table S2 (Supporting Information). The current is increased gradually as input voltage increases, and the power density also demonstrates a linear growth between current and voltage. Through parallel connection design, CBEF with larger sizes demonstrates outstanding and adaptable performance under varied energy inputs (Table S3, Supporting Information). Although the power efficiency decreased with enhanced DC input from excess heat loss, the CBEF evaporator still maintains outstanding electrothermal performance (Table S4, Supporting Information). In general, the CBEF evaporator demonstrates competitive electrothermal evaporation performance with or without the assistance of photothermal conversion compared with recent ESSG studies (Figure 4d).^[^ ^11^, ^13^, ^14^, ^15^, ^35^, ^39^, ^40^, ^41^, ^42^, ^43^ ^]^ After 8 cycles of operation (1 h per cycle), the average evaporation rate of CBEF‐P3 remains over 5.5 kg m^−2^ h^−1^ (Figure S26, Supporting Information), which is attributed to the robust framework and mechanical properties.

The redundant salt deposition due to poor salt‐rejection ability results in blocking the channel, destroying the evaporator structure, and weakening the efficiency.^[^ ^44^ ^]^ Therefore, the balance of efficiency and durability is critical for practical desalination. The desalination performance of CBEF‐P3 is investigated by simulating seawater with different concentrations. After 8 h of operation, the desalination rate under 1 kW m^−2^ radiation remains at ≈2.41, 2.99, 3.87, and 4.89 kg m^−2^ h^−1^ with 0, 1, 2, and 3 V DC inputs, respectively (Figure 4e). The slight decrease in the desalination rate compared with the pure‐water evaporation rate is caused by salt ions in brine, which delay the phase change from liquid to steam.^[^ ^45^, ^46^ ^]^ The desalination rate of CBEF‐P3 remains stable and strong after long‐term operation in 3.5 wt.% brine because of the outstanding hydrophilicity and water‐wicking performance of PPy@Tencel. The natural cracks on the fibers, the hollow channels inside the fibers, and hierarchical inter‐fiber gaps from core layer of twisted PPy@Tencels can provide abundant water pathways.^[^ ^37^ ^]^ Besides, the interconnected porous fibrous network from a unique core‐sheath structure facilitates ion migration and ion dilution to avoid nucleation of salt particles and the spontaneous ion exchange with bulk brine results in durable salt rejection performance.^[^ ^47^, ^48^ ^]^ The salt exchange is accelerated with the rapid brine flow, and the accumulated salt ions over the evaporator surface dissolve back into the bulk brine because of the concentration gradient.

Therefore, the CBEF‐P3 evaporator remains intact and clean after long‐term operation in 3.5 wt.% brine (Figure 4f). In addition, the desalination rate remains stable after 20 days of operation (8 h d^−1^) (Figure S27, Supporting Information). The imbalance between the steam evaporation and water replenishment rates leads to salt deposition with an excessive energy supply (3 V DC and 1 kW m^−2^ radiation) during long‐term operation in high‐concentration brine (10 wt.%). Therefore, a moderate electrothermal/photothermal supply (2 V DC and 1 kW m^−2^ radiation) is more applicable to reconcile efficiency and durability during high‐concentration brine desalination (Figure S28, Supporting Information). The desalination rate in 10 wt.% brine is calculated as ≈3.53 kg m^−2^ h^−1^. In this case, there is no salt crystallization on the CBEF even after a long period (24 h) of ESSG. This characteristic guarantees the adaptability and practicality of CBEF during severe desalination.

### 2.4 Outdoor Scalable Desalination Performance

**Figure 5:**
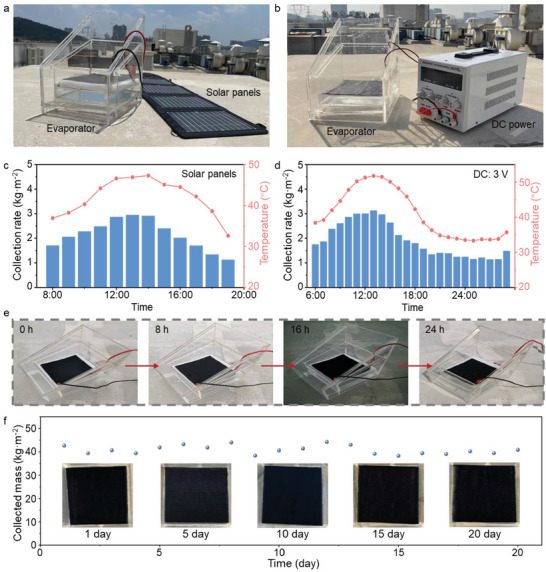
Outdoor scalable desalination performance of the CBEF evaporator. Digital image of the outdoor ESSG desalination device connecting a) solar panels and b) voltage supply. The outdoor evaporator temperature variation and freshwater collection rate of CBEF with c) solar panels and d) voltage supply on March 7–8, 2023, in Wuhan, China (113°41′ E, 29°58′ N). e) Digital images showing the morphology of the CBEF evaporator during the outdoor desalination process over 24 h. f) Photographs and ESSG performance of the CBEF evaporator during 20 days of desalination (8 h d^−1^).

The CBEF‐P3 evaporator with strong electrothermal/photothermal conversion demonstrates a synergistic desalination ability during the daytime and a continuous desalination ability overnight (Figure S29, Supporting Information). The outdoor experiment is conducted with a parallel‐connected large‐scale CBEF‐P3 (20 × 20 cm) evaporator connecting with solar panels and a DC supply, respectively (**Figure** **5**a,b). Seawater from the Yellow Sea is selected to investigate the practical performance in outdoor conditions (Wuhan, China. March 7–8, 2023) (Figure S30, Supporting Information). The purified water is collected using a water outlet under gravity after desalination (Figure S31, Supporting Information). The ion concentrations of the original seawater and the purified freshwater were characterized by ICP‐OES to examine the purification performance.^[^ ^38^, ^49^, ^50^ ^]^ The concentrations of four primary metal ions (Ca^2+^, Mg^2+^, Na^+^, and K^+^) decrease sharply after ESSG, far below the WHO standards for ion concentrations in drinking water (Figure S32, Supporting Information).^[^ ^51^, ^52^ ^]^

As shown in Figure 5c,d, the continuous electrothermal conversion makes the ESSG process sustainable and stable. Although the output voltage from solar panels is influenced by solar intensity, the CBEF evaporator also achieves a collection mass of 25.7 kg m^−2^ under 3.5 wt.% brine from 8:00 to 19:00. In addition, the outdoor daily collection mass is over 46 kg m^−2^ with a continuous 3 V DC input from the voltage supply. This weaker desalination rate compared to the laboratory experiment is attributed to array energy loss, weather variations, and saturated chamber humidity (Figure S33, Supporting Information). Even in this scenario, the daily desalination energy consumption per unit area is calculated as ≈1.29 kW h m^−3^, which is more cost effective than traditional desalination devices. In addition, the CBEF evaporator remains free of salt clogging after 24 h of desalination with continuous electrothermal/photothermal conversion (Figure 5e). The CBEF evaporator remains robust, and the EESG performance remains competitive during 20 days of consistent operation in severe outdoor conditions (Figure 5f).

For practical high‐concentration desalination (10 wt.%), an appropriate energy supply guarantees both efficiency and durability. The evaporator will not deteriorate with salt deposition after the whole day of operation (Figure S34, Supporting Information). The desalination collection of high‐concentration brine is still very considerable, and the energy consumption per unit area is ≈1.61 kW h m^−3^ (Figure S35, Supporting Information). Overall, the all‐weather‐available CBEF evaporator exhibits excellent efficiency, cost‐effectiveness, stability, and practicality.

## 3 Conclusion

In this work, an all‐weather‐available CBEF evaporator is fabricated by core‐sheath multilayer composite yarns through industrial production technology. The core/sheath PPy@Tencel layer ensures outstanding photothermal conversion and water circulation ability. The middle electric wire with customizable wrapping pitch and wrapping eccentricity enables optimized electrothermal conversion efficiency, heat distribution uniformity, and thermal transfer consistency. Herein, the evaporation rate of the CBEF evaporator under 3 V DC input 1 kW m^−2^ radiation is up to 5.57 kg m^−2^ h^−1^. The tunable energy supply guarantees operational adaptability during complex and severe desalination scenarios without salt deposition. The synergistic desalination ability during daytime and continuous desalination ability overnight ensures effective and stable long‐term operation. The average desalination energy consumption is only ≈1.61 kW h m^−3^ even in high‐concentration saline over the whole‐day operation. Thus, the CBEF evaporator provides a thorough investigation and analysis to enhance the electrothermal desalination efficiency and practicality. Cost‐effective desalination paves a novel and practical way for clean desalination and water purification.

## Conflict of Interest

The authors declare no conflict of interest.

## Author Contributions

X.D, C.G., Z.C., and Z.Z. contributed equally to this work. D.X., C.G., Q.Z., W.X., and J.F. conceived and planned this research. C.G., Z.Z., Z.C., and T.C. performed the experiments. Z.Z. performed the thermal modeling analysis. Q.Z., J.F., D.X., and C.G. organized the data and wrote the manuscript. All authors discussed the results and approved the final version of the manuscript.

## Supporting information

Supporting Information

## Data Availability

The data that support the findings of this study are available from the corresponding author upon reasonable request.
