# Supplementary material for: Photo‐Electro‐Thermal Textiles for Scalable, High‐Performance, and Salt‐Resistant Solar‐Driven Desalination
Source: Adv Sci (Weinh). 2024 Jun 19;11(31):2400623. doi: 10.1002/advs.202400623 (PMC11336979; doi:10.1002/advs.202400623)
Supplement: Supplementary file 1 — Supporting Information [file ADVS-11-2400623-s001.docx]

Supporting Information for

**Photo-electro-thermal textiles for s****calable, high-performance, and salt-resistant solar-driven desalination**

Duo Xu, ^‡abc^ Can Ge, ^‡ac^ Ze Chen, ^‡b^ Zhixun Zhang, ^‡b^ Qian Zhang, ^*b^ Tao Chen, ^b^ Chong Gao, ^b^ Weilin Xu, ^*b^ Jian Fang ^*ac^

^a^ College of Textile and Clothing Engineering, Soochow University, Suzhou 215123, China.

^b^ State Key Laboratory of New Textile Materials and Advanced Processing Technologies, Wuhan Textile University, Wuhan 430200, China.

^c^ National Engineering Laboratory for Modern Silk, Soochow University, Suzhou 215123, China.

E-mail: qzhang@wtu.edu.cn (Q.Z.); weilin_xu@wtu.edu.cn (W.X.); jian.fang@suda.edu.cn (J.F.)

‡ These authors contributed equally to this work.

**This PDF file includes:**

**Supplementary Materials**

Experimental Section Text 1.

Figures S1 to S35.

Tables S1 and S4.

**Experimental Section Text 1.**

**Materials**

Tencel yarn (19.7 tex) was purchased from Anhui Huamao Co. LTD. Commercial electric wire (nickel−chromium alloy) was purchased from WEISI Co. LTD. Pyrrole and ferric trichloride were purchased from Sinopharm Group Chemical Reagent Co. Ltd., China. Anhydrous ethanol was purchased from Aladdin.

**Manufacture of composite braiding electrothermal fabric**

**PPy@Tencel polymerization.** Tencel was first sonicated in anhydrous ethanol at 60 °C for 2 hours to remove impurities. The pretreated Tencel was dried in an oven for several hours. After that, the dried Tencel was immersed in a 0.18 mol/L ferric chloride solution for 0.5 h, and then Tencel was extracted and placed in a 0.15 mol/L pyrrole monomer solution for 2 h. Finally, the polypyrrole (PPy)-decorated Tencel yarn (PPy@Tencel) was achieved via polymerization.

**CBY fabrication.** First, multistranded PPy@Tencel yarns were converged at the braiding points to form core layer yarns using a high-speed braiding machine (16T-2, Xuzhou Henghui Braiding Machine Co., Ltd.) The PPy@Tencel yarns were wound onto the bobbin and mounted on the designated spindles. The reciprocating movement from one disk to another is beneficial for the interconnection between the PPy@Tencel yarns, and the number of spindles needed for winding the yarn depends on the number of feeding spindles. The high-speed braiding machine operated at a braiding speed of 15 rpm, winding speed of 2 m/min, and braided pitch of 60 mm. Second, the commercial electric wires of the middle layer were transferred from a bobbin to a hollow yarn bobbin through a yarn-pressing machine in a clockwise direction. After that, the core-layer PPy@Tencel was guided and fed into a hollow spindle (HKV141D-I covering machine, Zhejiang Jinggong Textile Technology Co., Ltd., China) with a tension controller. The core layer PPy@Tencel was then helically covered with commercial electric wires for high-speed wrapping to produce composite yarns. The wrapping pitches of commercial electric wires were controlled by the parameters of feeding speeds and wrapping speeds. Third, the sheath PPy@Tencel was secured to the outside yarn disc, and the fabricated composite yarns were wound onto a central bobbin and fed via a pretension device. The disc rotates the sheath PPy@Tencel around the double-layer composite yarns in both clockwise and counterclockwise directions to fabricate composite braiding yarn (CBY).

**CBEF fabrication.** The composite braiding electrothermal fabric (CBEF) was woven with nylon as warp yarns and CBY as weft yarns using an SGA598 semiautomatic weaving loom (Jiangyin Tongyuan Textile Technology Co., Ltd., China). Nylon yarns were used as tensional warp threads through the loom heddle, and CBYs were inserted as weft threads into the tensional warp to form a plain CBEF. For CBEFs, the total number of PPy@Tencel yarns was set to 24. When the number of core layer PPy@Tencel was set to 8, 10, 12, 14, and 16, the corresponding number of PPy@Tencel yarns in the sheath layer was set to 16, 14, 12, 10, and 8. The fabricated CBEFs were named CBEF-E1, CBEF-E2, CBEF-E3, CBEF-E4, and CBEF-E5 because the electric wires in the middle layer possess increased eccentricities. Correspondingly, the optimized CBEFs with decreased wrapping pitches of commercial electric wires in the middle layer were abbreviated as CBEF-P1, CBEF-P2, CBEF-P3, CBEF-P4, and CBEF-P5.

**Evaluation of evaporation performance.** The experiment was conducted at room temperature (approximately 24 °C), and the humidity was approximately 50 %. The real-time mass loss was monitored by an electronic balance (Precisa, XB2200C). The solar simulator was provided by a solar simulator (Beijing Perfectlight, PLS-FX300HU), and the solar intensity was calibrated with a light power meter (Beijing CEAulight Technology, CEL-NP2000).

The evaporation rate (ṁ) can be calculated by the equation:^[1]^

|  | $ṁ=\frac{\Delta m}{\mathrm{St}}$ | (1) |
| --- | --- | --- |

where Δm is the mass change of water during evaporation, S is the evaporation area, and t is the time of the evaporation process. That is, ṁ represents the mass change of evaporated water per unit area per unit of time.

Hence, the electrothermal efficiency (E) can be calculated by the following equation：^[2-4]^

|  | E=$\frac{h\cdot A(T_{1}-T_{0})R}{U^{2}}$ | (2) |
| --- | --- | --- |

where h is the convection heat transfer coefficient, and A means the electrothermal surface area. T_0_ and T_1_ are the stable surface temperatures before and after joule-heating, respectively. U refers to the applied DC voltage, and R denotes the resistance of the electrical wire.

Considering the synergistic effect between photothermal and electrothermal, the evaporation efficiency (η) can be calculated using the following equations ^[5-7]^:

|  | $\eta=\frac{\mathrm{Mh}_{v}}{C_{opt}P_{0}+Q}$ | (3) |
| --- | --- | --- |
|  | $Q=\frac{U\int Idt}{A}$ | (4) |

Where M is the specific evaporation rate, h_v_ means evaporation enthalpy, C_opt_ refers to the optical concentration on the absorber surface, P_0_ denotes the solar power density of 1 sun (1 kW·m^-2^), and Q is the heat obtained from the electrothermal process. Specifically, U is the voltage input, I represents the corresponding current, and A refers to the electrothermal area.

**The numerical simulation of electrothermal conversion.** The energy balance law was introduced to evaluate the saturation temperature of the heater. The saturated surface temperature is dependent on the applied voltage, material resistance, and heat transfer coefficient. The formula is as follows: ^[8, 9]^

|  | $\frac{U^{2}}{R}=mc\frac{\mathrm{dT}}{\mathrm{dt}}+hA({T-T}_{0})$ | (5) |
| --- | --- | --- |

where U is the applied voltage, R is the sheet resistance, c represents the specific heat capacity, m is the weight of the CBEF, T refers to the saturated surface temperature, T_0_ is the initial temperature, A is the electrothermal area, and h represents the heat transfer coefficient.

The details of simulation are as follows:

1. Building a geometric model. The diameters of electric wire and CBY are about 0.3 mm and 3 mm, respectively. The braiding eccentricities and pitches are set according to the actual sizes.

2. Defining the materials. The thermal conductivity, electric conductivity, and specific heat capacity of electric wire are set as 3.864 W·m^-1^·K^-1^, 2 μΩ·cm, and 0.39 kJ/(kg·℃), respectively.

3. Meshing and refinement. The total number of nodes and elements is 185,011 and 133,192, respectively.

4. Boundary and initial conditions. There is no thermal impedance between electric wire and yarn, the initial sample temperature is set as 25 ℃. A high potential is applied at the left end of the copper wire and a low potential at the right end for the simulation of electrothermal conversion. The surface heat exchange between the sample and environment has been considered, with an ambient temperature of 25 ℃ and a heat dissipation coefficient of 0.007.

**Characterization.** The morphology and microstructure of the samples were observed by a scanning electron microscope (FEI XL30, Sirion SEM) and optical microscope (RH2000, HIROX, Japan). The water contact angle was tested by a wettability meter (KONO SL200KS). The surface temperature was recorded by an infrared camera (FOTRIC 345) and thermocouple (SHSIWI TS-08A). The absorption and emission were tested by a UV‒VIS-NIR spectrophotometer (UV-2550, Shimadzu; Nicolet IS50) equipped with an integrated sphere. Fourier transform infrared (FTIR) spectroscopy was conducted using a spectrometer (Nicolet iS50, Thermo Scientific). The fabric's thermal conductivity was measured with a thermal conductivity meter (TPS 2500S, Shanghai K-Analysis Co., Ltd, China). In the outdoor experiment, water was taken from Dushu Lake and the Yellow Sea, China. The ion concentration was tracked by an ICP Spectrometer (ICAP 6000 SERIES, Thermo Scientific)

**Statistical Analysis.** Data were analyzed using Origin. Error bars generally represent the standard error of the mean and experiments were generally performed with a sample size of 3.


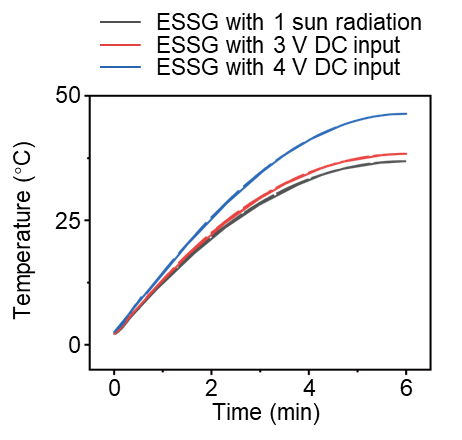


**Figure S1** The simulated surface temperature of the ESSG system with 1 sun radiation, 3 V DC input, and 4 V DC input.


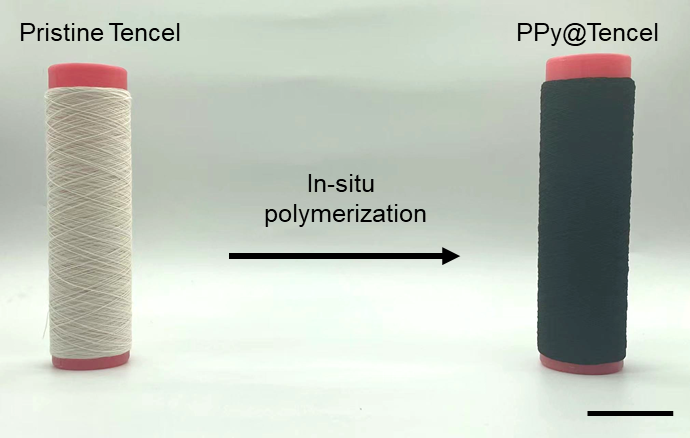


**Figure S2** Digital image comparison of PPy@Tencel morphology before and after in situ polymerization (scale bar: 5 cm).


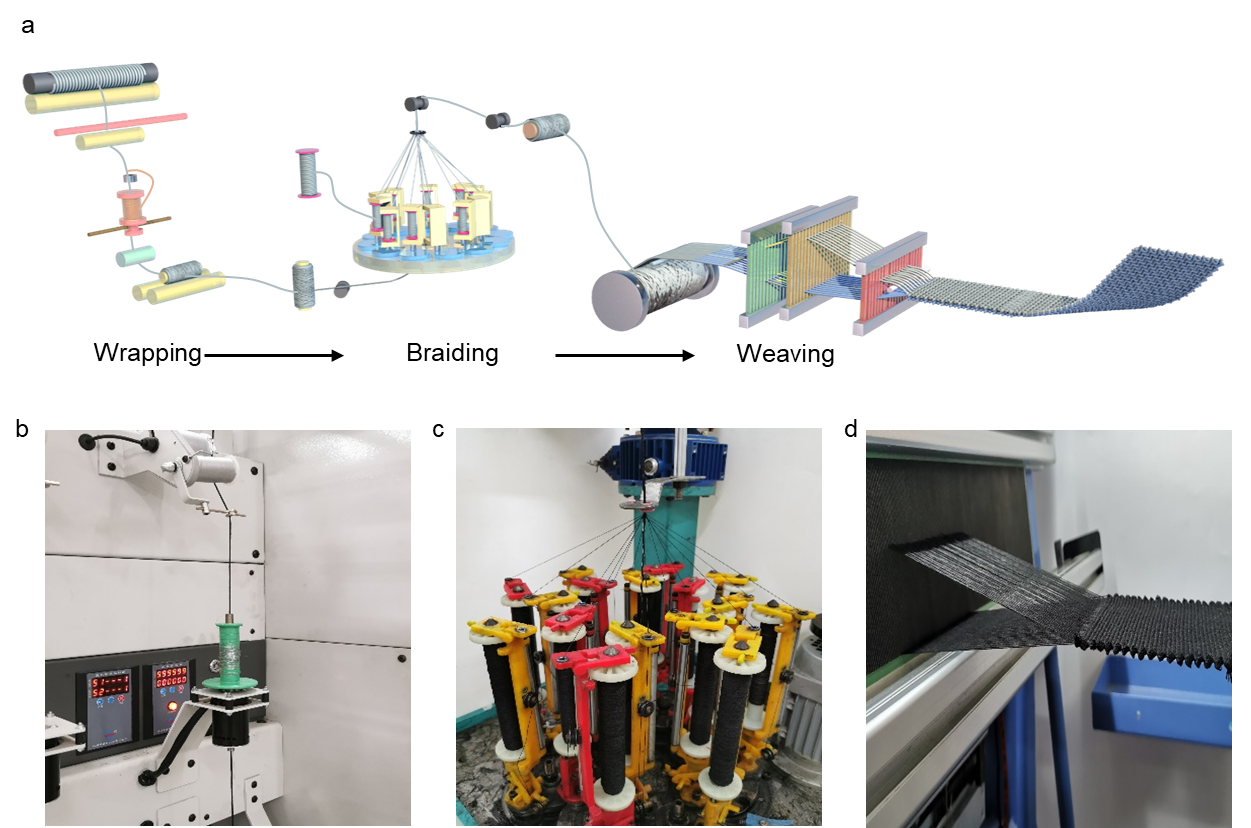


**Figure S3** Fabrication of CBEF evaporator. (a) Schematic drawing of the fabrication process of the CBEF evaporator. (b) The wrapping machine for wrapping electric wires with core PPy@Tencel. (c) The braiding machine for the fabrication of CBY. (d) The weaving machine for the fabrication of CBEF.


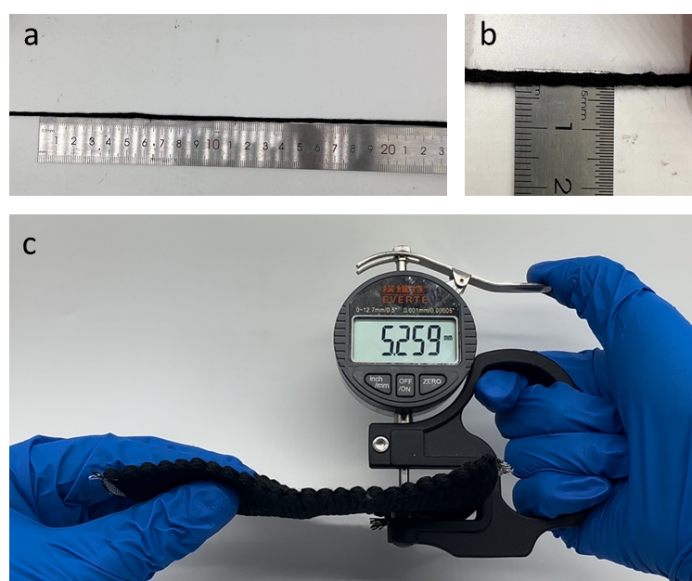


**Figure S4** Digital images showing the (a) CBY geometry, (b) CBY width, and (c) CBEF thickness.


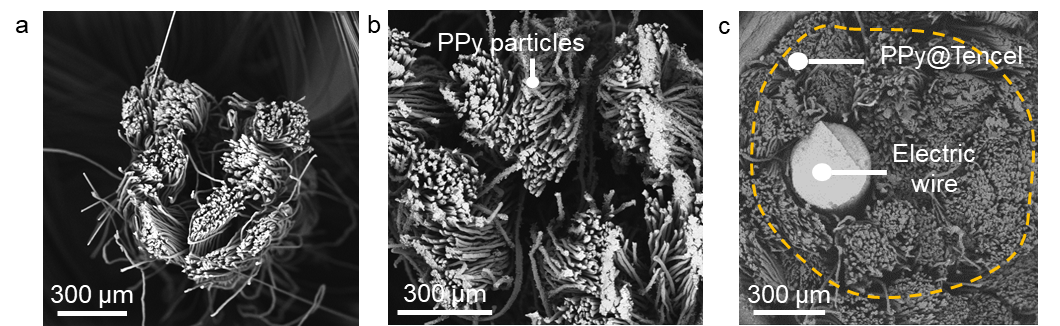


**Figure S5** SEM images of (a) pristine Tencel, (b) PPy@Tencel, and (c) CBY (Scale bar: 300 μm).


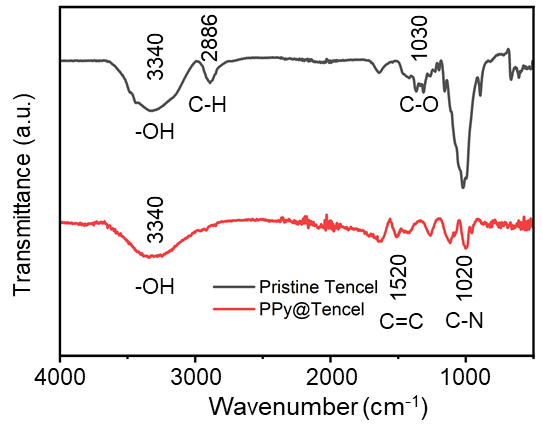


**Figure S6** FTIR spectra of pristine Tencel and PPy@Tencel samples.


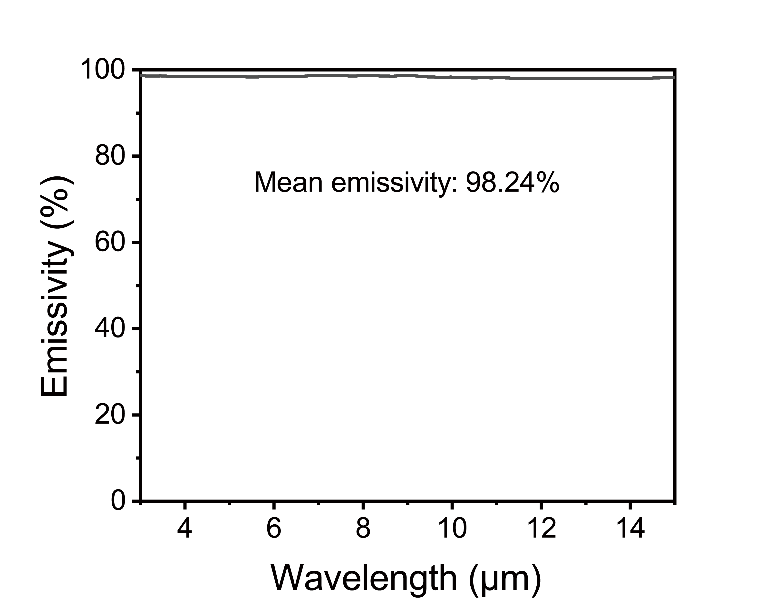


**Figure S7** The infrared emissivity of CBEF.


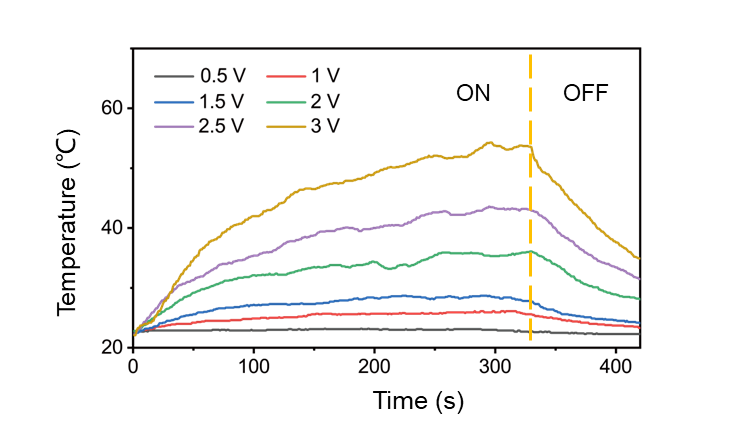


**Figure S8** Time-dependent dry temperature recording of CBY 10 cm in length under varied voltage inputs.


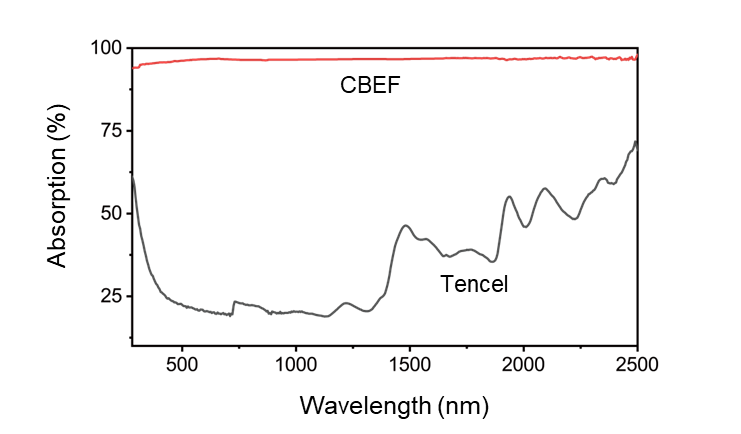


**Figure S9** Absorption spectra of the CBEF and pristine Tencel across the 280-2500 nm wavelength range.


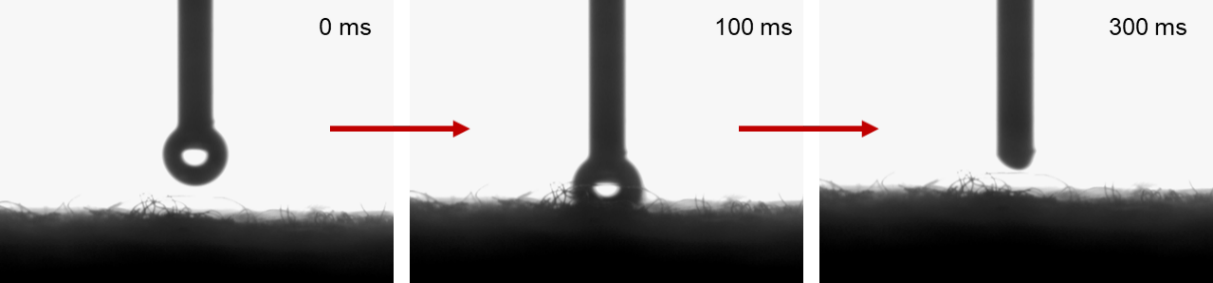


**Figure S10** Water contact angle test of CBEF.


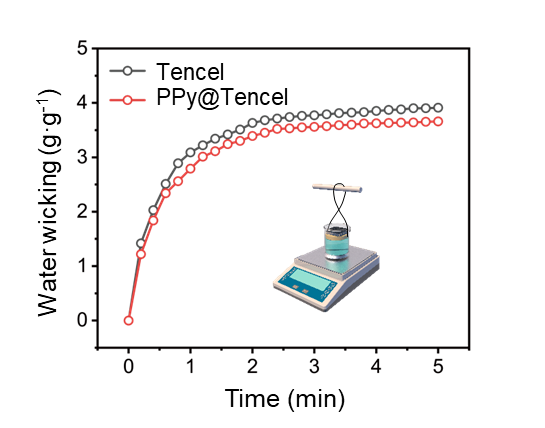


**Figure S11** Water wicking rate comparison of the PPy@Tencel and pristine Tencel over 5 min.


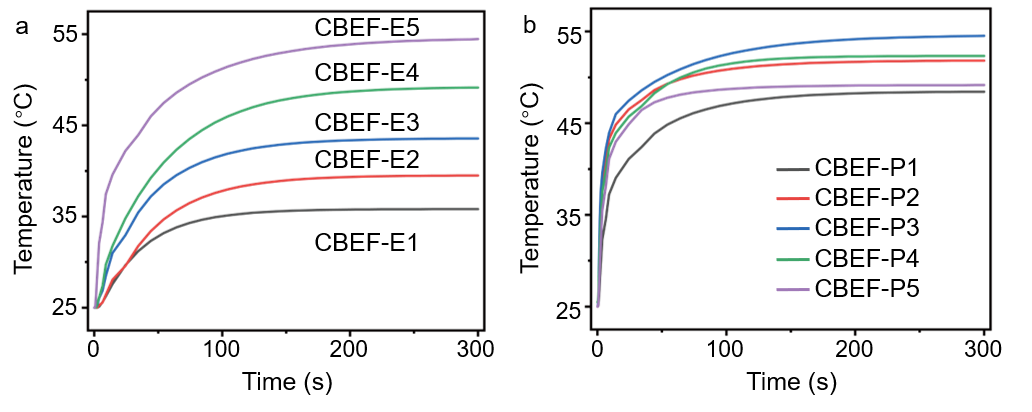


**Figure S12** Stimulated time-dependent dry temperature variations of (a) CBEF-Es and (b) CBEF-Ps samples with 3 V DC input.


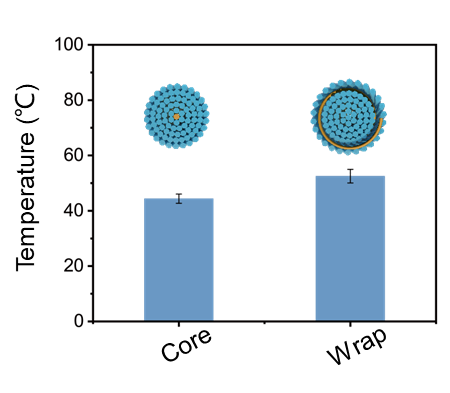


**Figure S13** Stable dry temperature comparison of composite yarns with electric wires as core yarns or wrap yarns.


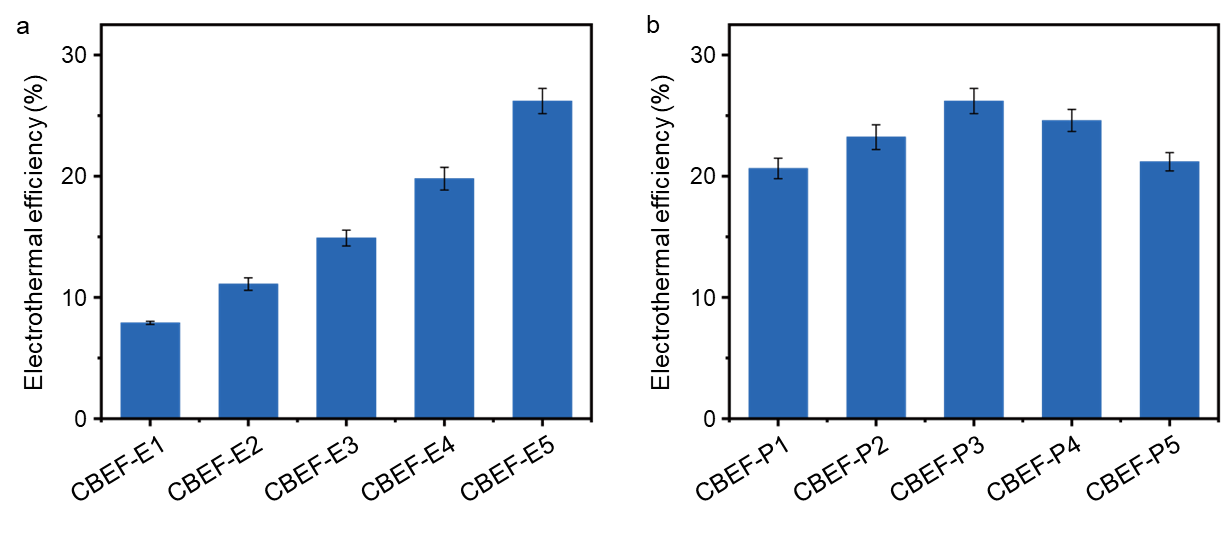


**Figure S14** The electrothermal efficiency of CBEF with various (a) wrapping eccentricity and (b) wrapping pitch under 3 V DC input.


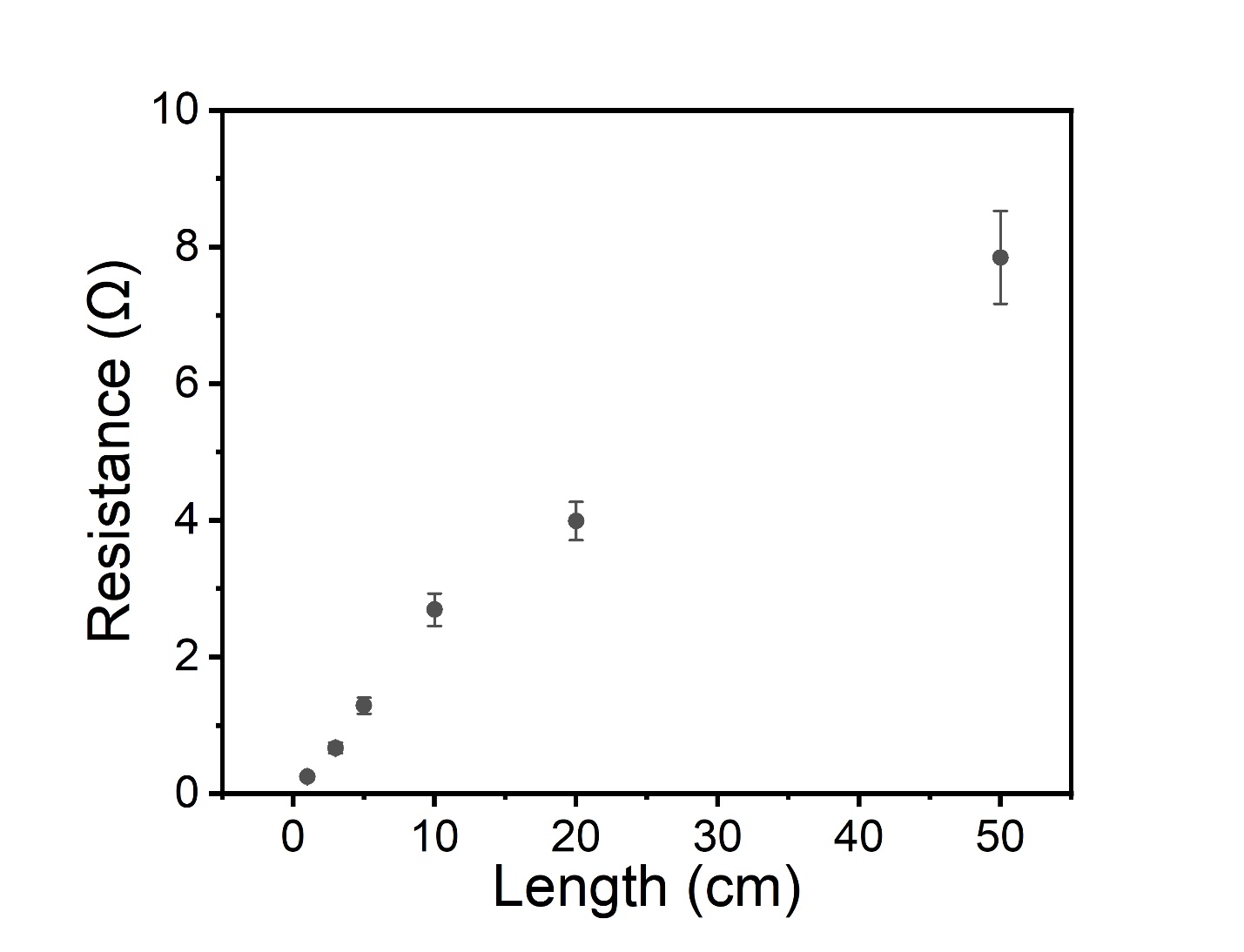


**Figure S15** The resistances of electric wires with different lengths.


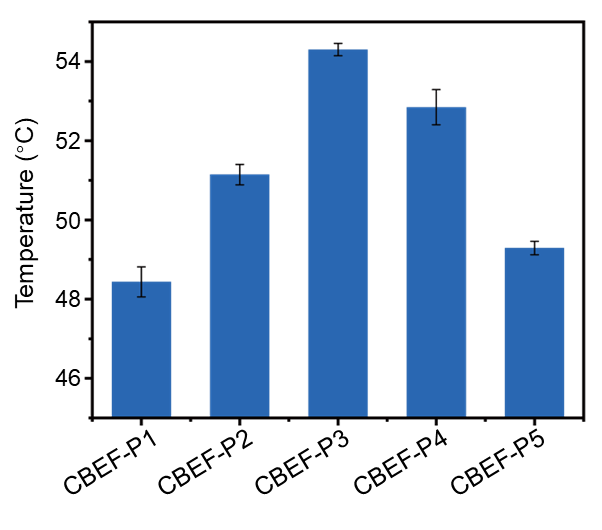


**Figure S16** The average stable dry temperature over the whole yarn section with 3 V DC input.


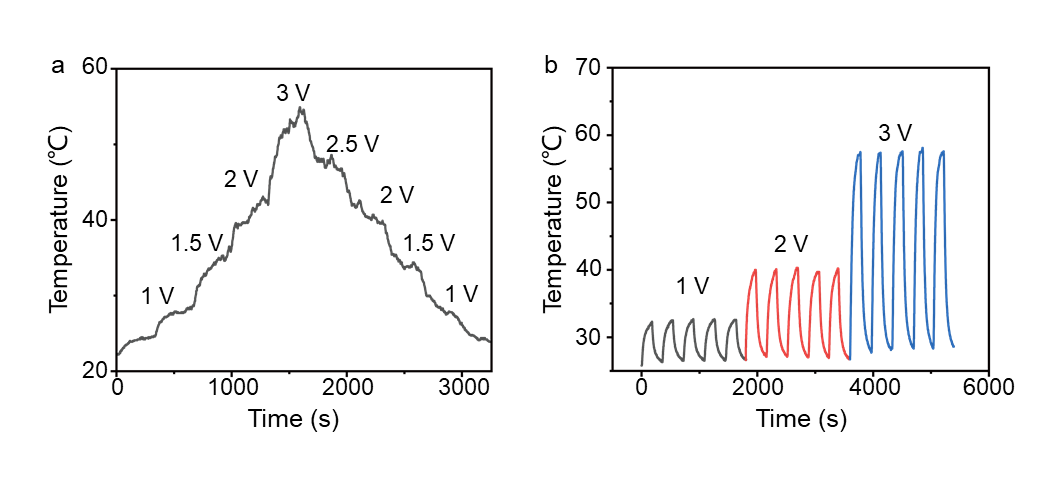


**Figure S17** The electrothermal response performance of CBEF-P3 under fluctuating voltage input.


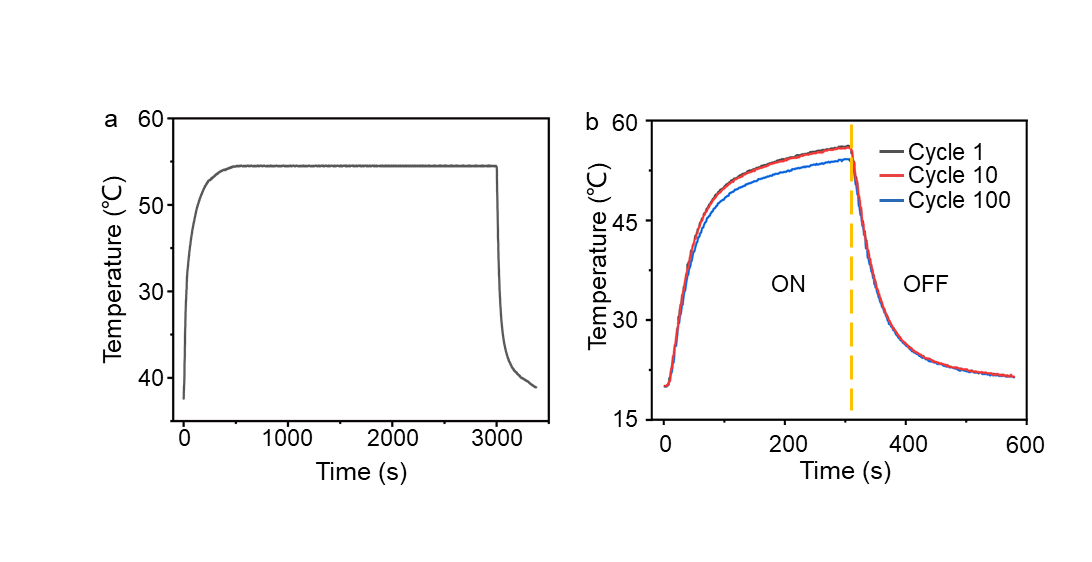


**Figure S18** The electrothermal stability and durability of CBEF-P3 under a 3 V voltage input.


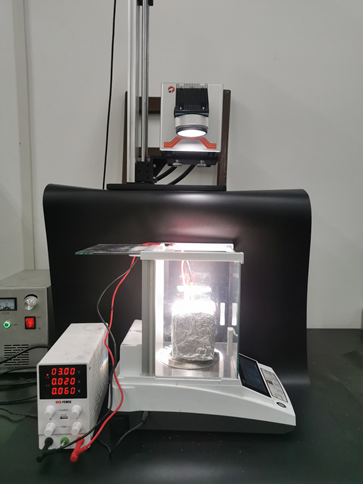


**Figure S19** Digital image of the ESSG test device.


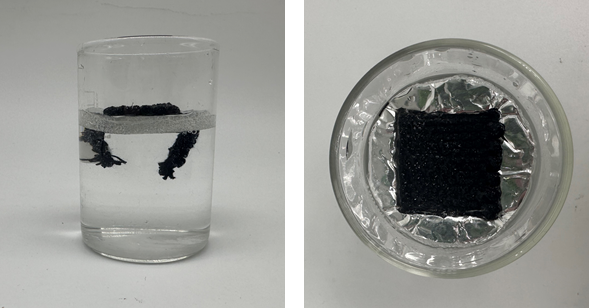


**Figure S20** Digital images showing the construction of the CBEF evaporator.


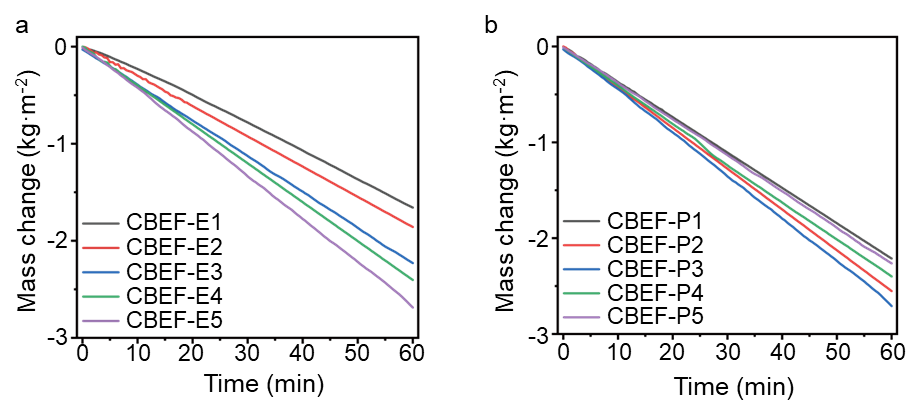


**Figure S21** Comparison of mass changes of (a) CBEF-Es and (b) CBEF-Ps over 60 min with 3 V DC input.


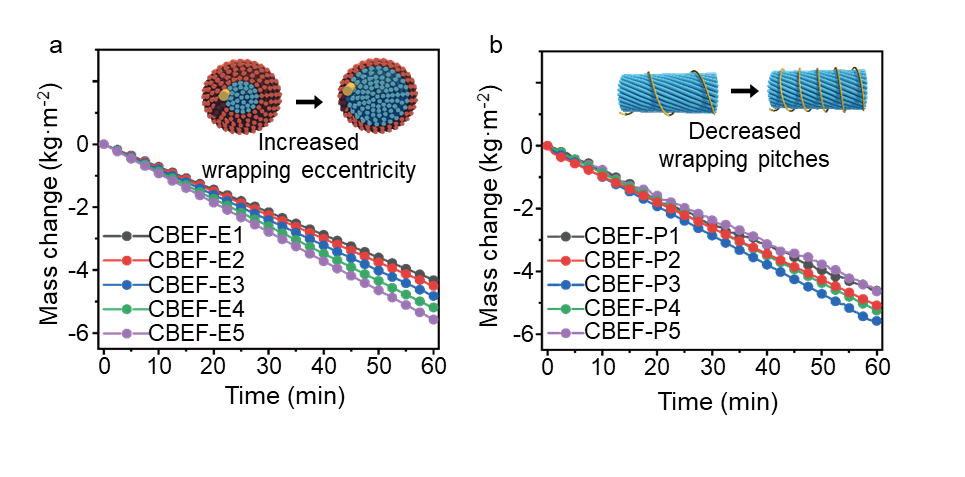


**Figure S22** Comparison of mass changes of (a) CBEF-Es and (b) CBEF-Ps over 60 min with 1 sun radiation and 3 V DC input.


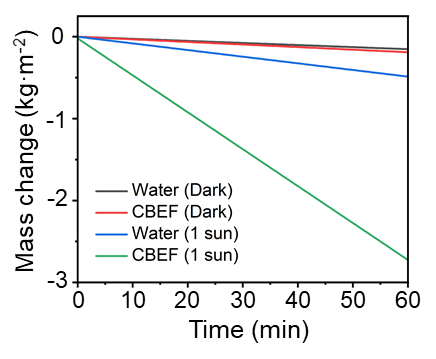


**Figure S23** Comparison of mass changes of pure water and CBEF with/without solar radiation.


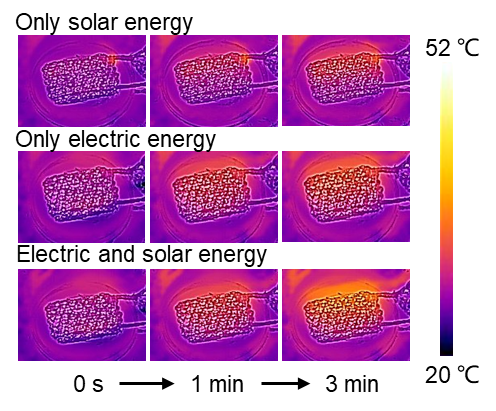


**Figure S24** Infrared images of the heating process of CBEF-P3 under different energy inputs.


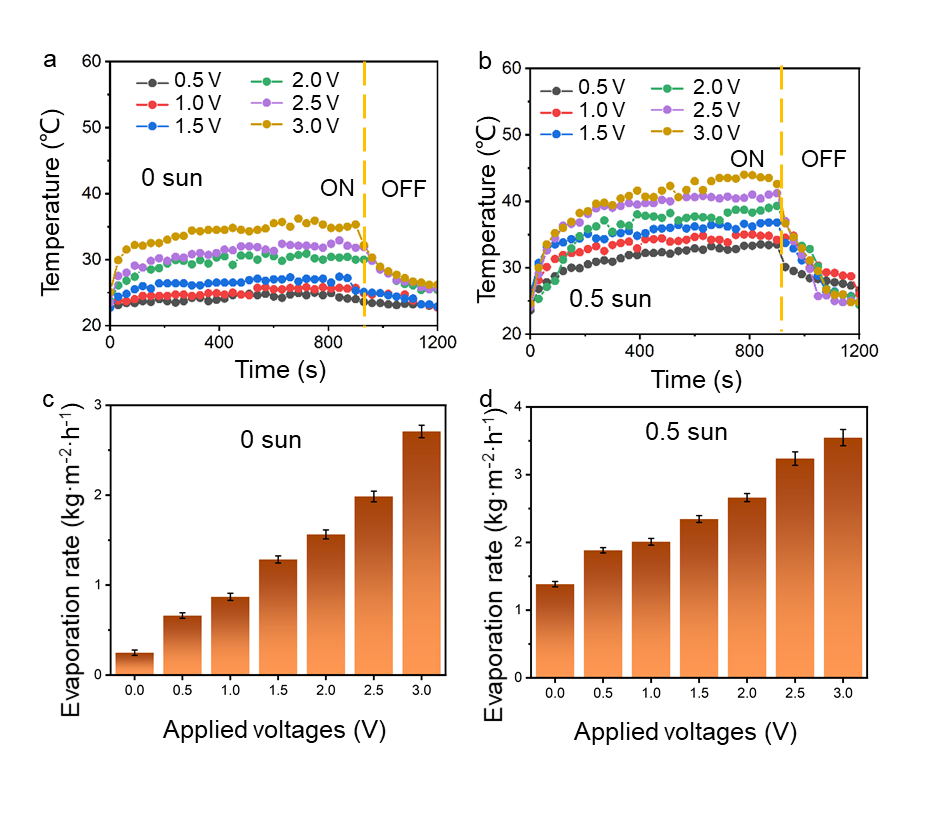


**Figure S25** Electrothermal coupled solar steam generation performance of the BCEF evaporator. Time-dependent wet temperature recording of CBEF-P3 under varied voltage inputs with the assistance of (a) 0 sun radiation and (b) 0.5 sun radiation. Evaporation rate of CBEF-P3 under varied voltage inputs with the assistance of (c) 0 sun radiation and (d) 0.5 sun radiation.


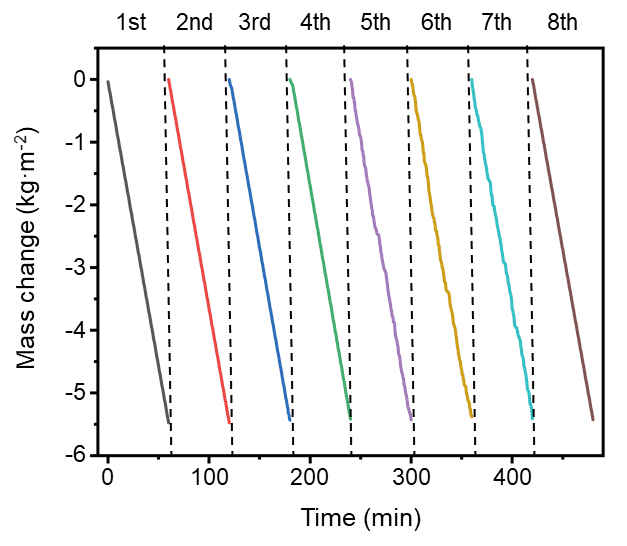


**Figure S26** Cycling stability of the time-dependent mass temperature change of CBEF-P3 under 1 sun illumination and 3 V DC input.


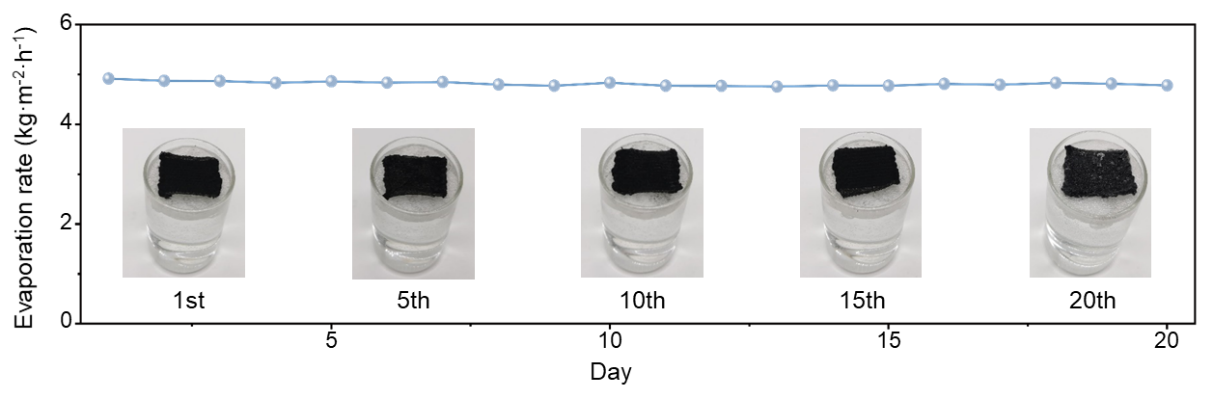


**Figure S27** Endurance test of the CBEF evaporator in a 3.5 wt% NaCl solution under 1 sun illumination and 3 V DC input (20 cycles, 8 h per cycle).


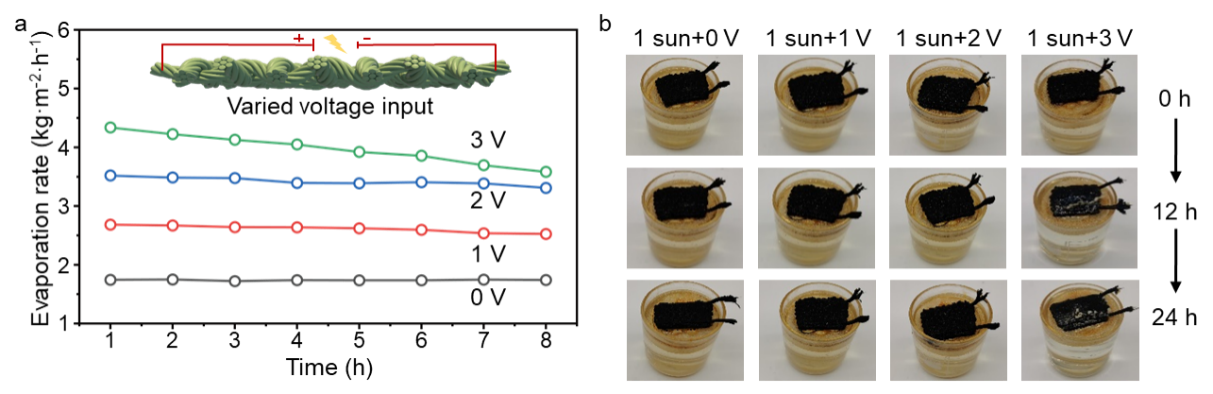


**Figure S28** The desalination performance of CBEF-P3 under 10 wt% brine. (a) The desalination rate comparison of CBEF-P3 with varied voltage inputs and 1 sun radiation. (a) Digital images showing the surface of CBEF-P3 during the whole-day desalination process with varied energy inputs.


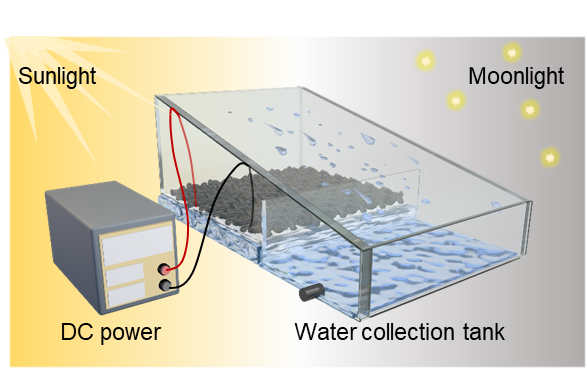


**Figure S29** Schematic diagram of the outdoor ESSG desalination device.


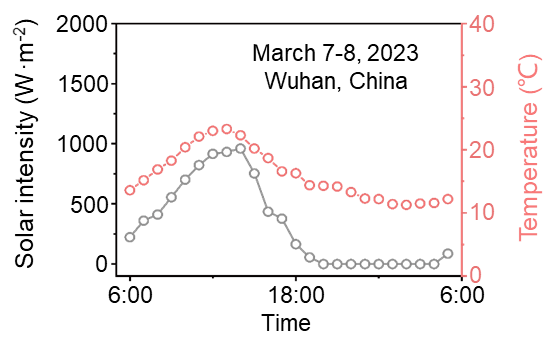


**Figure S30** Varying outdoor solar intensity and ambient temperature on March 7-8, 2023, in Wuhan, China (113°41′ E,29°58′ N).


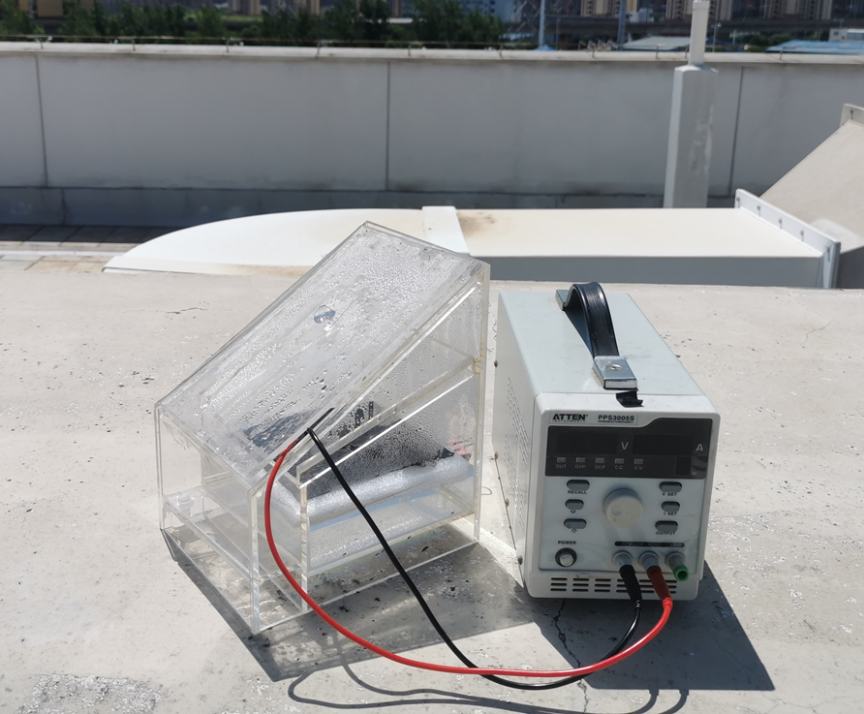


**Figure S31** The outdoor freshwater collection device of the ESSG system.


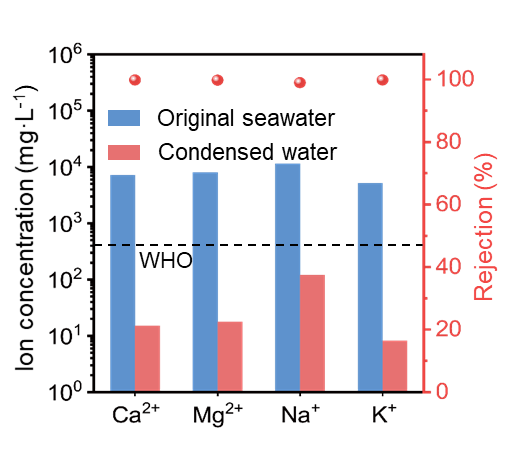


**Figure S32** Concentrations of four primary ions in seawater before and after desalination using a CBEF evaporator through outdoor ESSG.


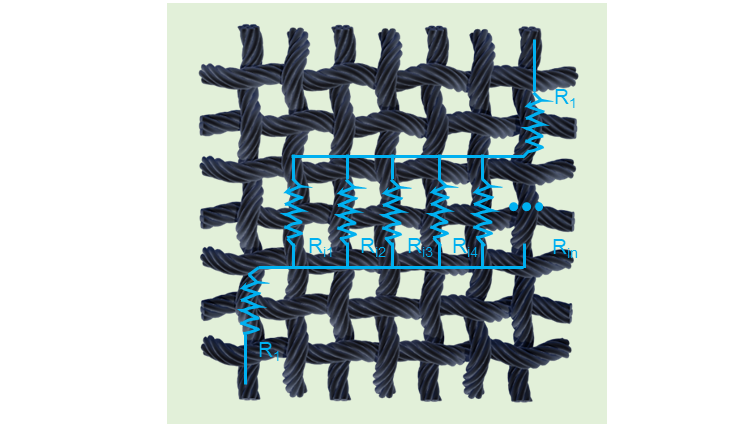


**Figure S33** Schematic diagram of circuits in series and parallel array connections.


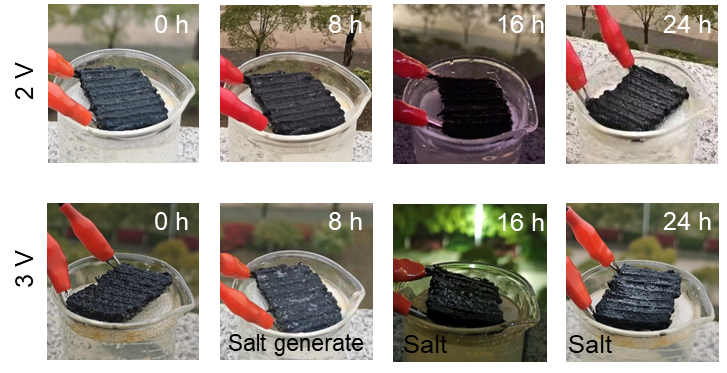


**Figure S34** Digital image recording of the continuous whole-day highly concentrated desalination process with different energy inputs.


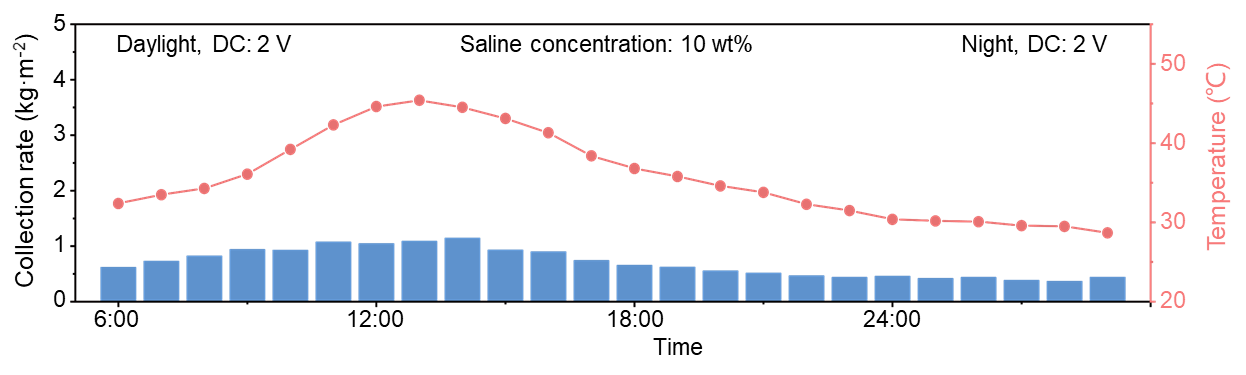


**Figure S35** The outdoor evaporator temperature variation and freshwater collection rate in high-concentration saline on March 7-8, 2023, in Wuhan, China (113°41′ E,29°58′ N).

**Table S1** The dimensions of CBY and CBEF samples.

| CBY  Width (mm) | CBY  Linear density (g/m) | CBEF  Thickness (mm) | CBEF  Surface density  (g/cm^2^) |
| --- | --- | --- | --- |
| 2.5±0.09 | 2±0.15 | 5.259±0.11 | 2.4±0.13 |

**Table S2** The electrothermal power densities of CBEF under varied voltage inputs.

| Voltage (V) | Current (A) | Area (cm^2^) | Power density (kW·m^-2^) |
| --- | --- | --- | --- |
| 0.5 | 0.7 | 12 | 0.29 |
| 1 | 1.23 | 12 | 1.02 |
| 1.5 | 1.53 | 12 | 1.91 |
| 2 | 1.6 | 12 | 2.67 |
| 2.5 | 1.95 | 12 | 4.06 |
| 3 | 2.28 | 12 | 5.7 |

**Table S3** The electrothermal power densities of CBEF with different sizes.

| Voltage (V) | Current (A) | Area (cm^2^) | Power density (kW·m^-2^) |
| --- | --- | --- | --- |
| 1 | 3.8 | 36 | 1.05 |
| 1 | 6.8 | 60 | 1.13 |
| 1 | 12.9 | 100 | 1.29 |
| 1 | 17.28 | 120 | 1.44 |

**Table S4** The evaporation performance under varied solar radiation and DC inputs.

| Voltage (V) | Power density (kW·m^-2^) | Solar radiation  (kW·m^-2^) | Specific evaporation rate (kg·m^-2^·h^-1^) | Evaporation efficiency (%) | Energy consumption  (kW·m^-2^) |
| --- | --- | --- | --- | --- | --- |
| 1 | 1.02 | 0 | 0.45 | 29.9 | 0.31 |
| 2 | 2.67 | 0 | 0.81 | 20.7 | 0.55 |
| 3 | 5.7 | 0 | 1.41 | 16.8 | 0.95 |
| 0 | 0 | 0.5 | 0.71 | 97.8 | 0.49 |
| 1 | 1.02 | 0.5 | 1.04 | 46.6 | 0.71 |
| 2 | 2.67 | 0.5 | 1.38 | 29.7 | 0.94 |
| 3 | 5.7 | 0.5 | 1.84 | 20.2 | 1.25 |
| 0 | 0 | 1 | 1.38 | 93.7 | 0.93 |
| 1 | 1.02 | 1 | 1.69 | 56.7 | 1.14 |
| 2 | 2.67 | 1 | 2.17 | 40.2 | 1.47 |
| 3 | 5.7 | 1 | 2.89 | 34.4 | 1.96 |

**Reference**

1. Z. Chen, Q. Li, X. Chen, *ACS Sustain. Chem. Eng.* **2020,** *8*, 13850-13858.

2. X. Meng, W. Xu, Z. Li, J. Yang, J. Zhao, X. Zou, Y. Sun, Y. Dai, *Adv. Fiber Mater.* **2020,** *2*, 93-104.

3. F. Yang, J. Chen, Z. Ye, D. Ding, N. V. Myung, Y. Yin, *Adv. Funct. Mater.* **2020,** *31*, 2006294.

4. G. Cheng, X. Z. Wang, X. Liu, Y. R. He, B. V. Balakin, *Sol. Energy* **2019,** *194*, 415-430.

5. X. Z. Wang, Y. R. He, X. Liu, G. Cheng, J. Q. Zhu, *Appl. Energy* **2017,** *195*, 414-425.

6. T. Li, Q. Fang, J. Wang, H. Lin, Q. Han, P. Wang, F. Liu, *J. Mater. Chem. A* **2021,** *9*, 390-399.

7. M. Wang, W. He, Y. Hua, X. Xie, S. Chen, L. Zhou, Y. Zhang, Y. Hou, S. Lin, H. Xia, J. Zheng, X. Hou, *SusMat* **2022,** *2*, 679-688.

8. P. Du, J. Zhang, Z. Guo, H. Wang, Z. Luo, Z. Fan, B. Li, Z. Cai, F. Ge, *J. Mater. Sci. Technol.* **2022,** *122*, 200-210.

9. D. Janas, K. K. Koziol, *Nanoscale* **2014,** *6*, 3037-3045.
